# Colorectal Oncogenesis and Inflammation in a Rat Model Based on Chronic Inflammation due to Cycling DSS Treatments

**DOI:** 10.1155/2011/924045

**Published:** 2011-10-05

**Authors:** Åsa Håkansson, Camilla Bränning, Göran Molin, Diya Adawi, Marie-Louise Hagslätt, Margareta Nyman, Bengt Jeppsson, Siv Ahrné

**Affiliations:** ^1^Food Hygiene, Division of Applied Nutrition and Food Chemistry, Lund University, 221 00 Lund, Sweden; ^2^Division of Applied Nutrition and Food Chemistry, Lund University, 221 00 Lund, Sweden; ^3^Department of Surgery, Skåne University Hospital, Lund University, 205 02 Malmö, Sweden

## Abstract

Inflammation is known to be linked with development of colorectal cancer, and the aim was to assess the malignant potential and degree of inflammation in a dextran-sulphate-sodium-(DSS-) induced cyclic colonic tumour model (CTM) in rats and to compare it with the azoxymethane-(AOM-) induced CTM model. Tumours developed in both groups, although, in the DSS group, the colonic mucosa appeared edematous and the number of haemorrhagic erosions and quantity of dysplastic lesions were higher as well as the mucosal concentration of myeloperoxidase and faecal viable count of *Enterobacteriaceae*. The livers were affected as evaluated by steatosis, parenchymal loss, haemorrhage, and inflammatory infiltrations, and higher proportions of acetate and lower proportions of butyrate in colonic content were found. The DSS model seems to mimic the clinical situation and may be valuable for investigation of inflammation-related dysplasia and colon cancer, as well as for altered liver function by endogenous inflammatory mediators.

## 1. Introduction

Chronic inflammation is characterised by a continued active inflammatory response and tissue destruction, and it seems to be a driving mechanism for promoting the development of carcinoma in colon and rectum of patients suffering from ulcerative colitis (UC), one of the major forms of the idiopathic inflammatory bowel diseases [1]. Compared to the general population, long-term UC patients have high risk of developing colorectal cancer, which increases as the extent and duration of the disease increase [2]. 

Mucosal inflammation may lead to colonic carcinogenesis through different mechanisms, such as induction of genetic mutations, increased cryptal cell proliferation, changes in crypt cell metabolism and bile-acid enterohepatic circulation, and alterations in bacterial flora [3, 4]. Dysplasia describes architectural and cytological abnormalities in the epithelium that predispose an organ to cancer development, and dysplasia has been indicated as an indicator of malignancy in UC [5]. The colonic epithelium provides a critical barrier to protect the host from resident commensal and pathogenic microbes. Inflammation within the gastrointestinal tract profoundly influences mucosal integrity and its ability to resist injury induced by luminal factors. In patients with adenocarcinoma of the large bowel, disruption of the intestinal barrier is assumed, and findings suggest that intestinal bacteria translocate from the bowel in large numbers [6]. 

The microbiota of the distal ileum and colon are complex, metabolically active and interact with intestinal epithelial and immune cells. Compositional changes in the intestinal microbiota can lead to decreased protective and increased quantities of aggressive bacterial species, and more proinflammatory faecal microbial communities have been observed in patients with UC compared with healthy individuals [7]. Lipopolysaccharide (LPS), derived from the outer envelope of Gram-negative bacteria that are physiologically part of the gut microbiota, is a major inducer of the inflammatory response and causes extensive damage to a variety of organs, including the liver [8], during increased intestinal permeability and bacterial translocation. A potential link between bacterial components and hepatobiliary inflammation has been substantiated [9], and a wide range of hepatic histological abnormalities has been found in patients with chronic UC [10], with fatty infiltration of hepatocytes and primary sclerosing cholangitis being the most common lesions [11]. 

Short-chain fatty acids (SCFAs), produced in the colon during anaerobic fermentation by the microflora, represent a major constituent of the luminal contents. The three major SCFAs formed during fermentation are acetate, propionate, and butyrate, among which butyrate and propionate most efficiently ameliorate an ongoing inflammatory response [12].

Mechanisms of colon carcinogenesis have been elucidated using several animal models, the majority using azoxymethane (AOM) or other carcinogenic agents alone or in combination with dextran sulphate sodium (DSS). DSS is a nongenotoxic sulphated polysaccharide used to induce experimental chronic colitis and colitis-associated neoplasia, histopathologically reminiscent of human UC [13], but the precise mechanisms by which DSS induces colonic inflammation are still unknown. As observed in humans, dysplasia and/or cancer develop as flat lesions or as dysplasia-associated lesions during long-term DSS administration [14]. AOM is a colon-specific carcinogen that serves as an effective tool for assessing colon tumours in susceptible rodents [15]. Administration of AOM is an extremely efficient method of inducing adenocarcinoma in the colon. However, whether or not this model represents inflammation-driven carcinogenesis can be questioned.

It is therefore of interest to gain insight into the relationship between colonic inflammation and carcinogenesis in a model with prolonged inflammatory stress. In the present study, we have focused on histopathological evaluation of colon and liver specimens, *Enterobacteriaceae* versus lactobacilli, SCFAs, and inflammatory markers.

## 2. Materials and Methods

### 2.1. Animals and Experimental Design

Female Sprague-Dawley rats were obtained from Möllegård (Viby, Denmark), and they were housed four per cage in plastic-bottomed cages. Animals were allowed free access to water, while feed intake was restricted to 23 g (dwb, dry weight basis) per rat and day. Lighting was controlled on a 12-h light-dark cycle and temperature maintained at 22°C. All procedures involving animals and their care were approved by the Ethics Committee for Animal Studies at Lund University. Rats were divided into three groups: untreated animals, that is, the normal control (NC group), animals treated in cycles with dextran sulphate sodium (DSS group), and animals treated with azoxymethane (AOM group). The DSS group was administered 4% (w/v) DSS (MW = 36,000–50,000; ICN Biomedicals Inc., Aurora, Ohio) dissolved in drinking water for 7 days, followed by 10 days of tap water, and this cycle was then repeated 11 times. 

The DSS solution was changed daily. The AOM group was given a single intraperitoneal injection (15 mg/kg bodyweight) of AOM (Sigma, St. Louis, USA, dissolved in 0.9% NaCl), and starting 1 week after the injection, animals were administered 5% DSS in drinking water for 7 days, after which the rats were monitored without further treatment for 18 weeks. All chemicals were of analytical grade. 

Throughout the study, the diet to all rats included oat bran at a level of 50 g dietary fibre/kg (dwb) (Table 1). The dry matter content was adjusted with wheat starch, and the content of dietary fibre was 17.2 g/100 g (dwb), where 1.6 g/100 g (dwb) was Klason lignin, that is, components not soluble in 12 M H_2_SO_4_. The nonstarch polysaccharides consisted mainly of glucose (61%), xylose (19%), and arabinose (12%) (data not shown). After 7 days of adaptation to the diet, the experimental period started and feed residues were collected daily.

Rats were weighed before and after the adaptation period, as well as daily during the DSS consumption. An attempt was made to quantify the amount of drinking water and DSS load ingested by the rats. Drinking volumes were recorded every 24 h for each cage (four animals), and the DSS load per animal was calculated over the experimental period as: (total drinking water (mL) × (DSS (g)/100 mL))/number of animals.

### 2.2. Sampling

Blood samples for analysing haptoglobin and SCFAs were taken from the saphenous vein at the beginning of the study for all groups, during cycle 1, 5, and 10 for the DSS group and for the AOM and NC groups, at the same sampling times as for the DSS group. During each DSS cycle, samples were taken on the seventh day of DSS administration and on the tenth day of the following water period. At the same time, faecal samples were collected for viable count and body temperature was measured.

At the end of the experiment, the animals were anaesthetised with Hypnorm (Division of Janssen-Cilag Ltd., Janssen Pharmaceutica, Beerse, Belgium), Dormicum (F. Hoffman-La Roche AG, Basel, Switzerland), and water (1 : 1 : 2) at a dose of 0.15 mL/100 g of body weight by a subcutaneous injection. For analysis of cytokines and SCFAs, arterial blood was collected and the mesenteric lymph nodes and liver were obtained for bacterial translocation and liver histology. The entire colorectum from the colocaecal junction to the anal verge was excised, the luminal content of caecum and colon was gently removed for analysis of SCFAs, and pH was measured in caecal content before storage at −40°C. The large bowel was macroscopically examined for gross lesions all of which recorded, and then the colon was cut and fixed in 10% buffered formalin, for 24 hr. Histological examination was performed on paraffin-embedded sections, after haematoxylin and eosin (H&E) staining. Tissue samples from distal colon were also stored for analysis of myeloperoxidase (MPO).

### 2.3. Clinical Scoring of Colitis

DSS-induced disease severity was analysed in terms of disease activity index (DAI), which in turn was calculated on the basis of weight loss, stool consistency, and rectal bleeding. The scoring system has been validated [16] and shown to correlate histologically with pathological findings [13]. The DAI was assessed daily from day 0 to day 7 and scored on a scale of 0–4 for each clinical parameter and then averaged for each animal. Weight-loss, stool, and bleeding scores were defined by modified scoring limits [17].

### 2.4. Myeloperoxidase (MPO) Activity

Specimens of distal colon were collected for measurement of myeloperoxidase (MPO) and weighed prior to storage at −70°C until time of assay. The assay procedure was done in accordance with Osman et al. [18]. The activity was expressed as units per gram of wet weight of the tissue.

### 2.5. Histological Evaluation

Specimens from the distal part of colon and liver were evaluated by light microscopy. Evaluation of macroscopic abnormalities through the entire length of colon and microscopic alterations was evaluated by an experienced surgeon and pathologist, respectively. The biopsy sites of the distal colon, taken at chosen sampling sites (polyps or dysplastic lesions and surrounding mucosa), and the left lobe of the liver were each fixed in neutral buffered formalin, followed by standard procedure for paraffin embedding. Serial sections were cut for each organ and stained with haematoxylin-eosin staining. The histological images are chosen to show different findings in the different groups and do not illustrate the condition of the complete biopsy. In the colon, the degree of dysplasia was scored from normal mucosa to mucosa with mild dysplasia (with distorted crypts of abnormal length and orientation) and severe dysplasia (with severe crypt distortion, atypical epithelial cells, reduction or loss of goblet cells, hyperchromatic cell nuclei, and increased numbers of cell mitoses). A numerical scoring system was applied to enable statistical evaluation (1 = normal mucosa; 2 = low grade dysplasia; 3 = high grade dysplasia).

Liver specimens were evaluated for the degree of steatosis according to Brunt et al. [19], where steatosis was scored as absent (= 0), mild when present in <1/3 of the hepatocytes (= 1), moderate when present in 1/3-2/3 of the hepatocytes (= 2), and severe when present in >2/3 of the hepatocytes (= 3). The presence and location of infiltrating inflammatory cells and liver injury were also recorded. Degree of inflammation in steatotic and nonsteatotic areas, stasis, and loss of parenchyma were graded using a semiquantitative scale of 0 (absent), 1 (mild), 2 (moderate), and 3 (extensive) [20].

### 2.6. Bacterial Translocation

To measure bacterial translocation across the intestinal epithelium, samples from the caudate lobe of the liver and mesenteric lymph nodes were collected aseptically and frozen immediately at −70°C until determination. After thawing, samples were placed in an ultrasonic bath (Millipore, Sundbyberg, Sweden) for 5 min and swirled on a Chiltern for 2 min. Viable counts were obtained from Violet-Red Bile Glucose (VRBG) agar (Oxoid) that was incubated aerobically at 37°C for 24 h (*Enterobacteriaceae *count), Brain Heart Infusion (BHI) agar (Difco, Detroit, Mich) that was incubated aerobically and under anaerobic condition, as described above, at 37°C for 72 h (aerobic and anaerobic bacterial count, resp.), and from Rogosa agar (Oxoid), incubated anaerobically at 37°C for 72 h (lactobacilli count). Results were expressed as incidences of positive cultures/group.

Colonies were randomly picked from the plates with positive cultures, and the isolates were subcultured before subjected to 16S rDNA sequencing.

### 2.7. Viable Count of *Enterobacteriaceae* and Lactobacilli in Faeces

Faecal samples were thawed and homogenised in the freezing medium, diluted, and plated on Rogosa agar for lactobacilli count (Oxoid; incubated anaerobically (Gas Pack System, Gas Pack; Becton Dickenson Microbiology Systems, Cockeysville, Md) at 37°C for 72 h) and VRBG for *Enterobacteriaceae* count (Oxoid; incubated aerobically at 37°C for 24 h). 

Colonies were randomly picked from countable Rogosa agar plates (10–150 colonies). Altogether, 44 isolates were collected.

### 2.8. Randomly Amplified Polymorphic DNA (RAPD) Analysis

As template for the polymerase chain reaction, crude cell extract was prepared in accordance with the protocol of Quednau et al. [21] and one microlitre of PCR template was used in the polymerase chain reaction (PCR) [21]. Agarose gel (Type III, High EEO, Sigma) electrophoresis was run, and the gels were stained with ethidium bromide and photographed under UV illumination.

### 2.9. 16S rDNA Sequencing

The primers used for amplification of the 16S rRNA genes were ENV1 (5′-AGA GTT TGA TII TGG CTC AG-3′, *Escherichia coli* numbering 8–27) and ENV2 (5′-CGG ITA CCT TGT TAC GAC TT-3′, *E. coli *numbering 1511–1492) [22]. The PCR reaction mixture contained 0.2 *μ*M of both primers, 5 *μ*L of template DNA, 5 *μ*L of 10x PCR reaction buffer with 1.5 mM MgCl_2_ (Roche Diagnostics GmbH, Mannheim, Germany), 200 *μ*M of each deoxyribonucleotide triphosphate, and 2.5 U of Taq DNA polymerase (Roche Diagnostics, Mannheim, Germany) in a final volume of 50 *μ*L. PCR was performed in a PCR Mastercycle 5333 (Eppendorf) with the following profile: 1 cycle at 94°C for 3 min, followed by 30 cycles of 96°C for 15 s, 50°C for 30 s, and 72°C for 90 s, with an additional extension at 72°C for 10 min. PCR products (5 *μ*L) were verified on 1.5% (wt./vol.) agarose gel in 1x TBE buffer (89 mM Tris, 89 mM boric acid, 2.5 mM EDTA, pH 8.3), after ethidium bromide staining. Amplicons were single strand sequenced by MWG (Biotech, Ebersberg, Germany), and the 16S rDNA sequences (mostly around 500 bp) were subjected to BLAST search against GenBank [23] or aligned to 16S rDNA encoding sequences retrieved from the Ribosomal Data Base (RDP-II) [24] for an approximate phylogenetic affiliation.

### 2.10. Dietary Fibre

The soluble and insoluble dietary fibres in oats were determined by a gravimetric method [25]. The composition of the fibre residues was analysed by gas-liquid chromatography (GLC) for the neutral sugars as their alditol acetates and spectrophotometrically for the uronic acids [26].

### 2.11. Short-Chain Fatty Acids (SCFAs)

The SCFAs (acetic, propionic, isobutyric, butyric, isovaleric, and valeric acids) were analysed in serum using GLC [27] with small modifications. Water and 2-ethylbutyric acid (internal standard) were added to the serum samples, and the SCFAs were protonised with hydrochloric acid. To enrich the SCFAs, a hollow fibre was immersed in the serum solution and the SCFAs were extracted into the lumen of the fibre. After 16 h of extraction, the lumen content was flushed and mixed with hydrochloric acid before being injected onto a fused-silica capillary column (DB-FFAP 125-3237, J&W Scientific, Agilent Technologies Inc., Folsom, Calif, USA). ChemStation software (Agilent Technologies Inc., Wilmington, Del, USA) was used for the analysis GC. 

The caecal and colonic amounts of SCFAs (acetic, propionic, isobutyric, butyric, isovaleric, valeric, caproic, and heptanoic acids) were analysed by a GLC method [28] with minor modifications. Water mixed with hydrochloric and 2-ethylbutyric acids were added to the faecal samples. The suspensions were homogenised with an Ultra Turrax T25 basic (IKA-WERKE, Staufen, Germany) and then centrifuged (MSE Super Minor, Hugo Tillquist AB, Solna, Sweden) before injection onto a fused-silica capillary column (see earlier).

### 2.12. Body Temperature

Body temperature of each rat was measured with a rectal digital thermometer at baseline and after the administrations of DSS and the subsequent water period at cycle 1, 5, and 10 and during corresponding points in time for NC and AOM groups. The rats were placed in a restraining device when the body temperature was measured.

### 2.13. Haptoglobin

The concentration of serum haptoglobin was analysed using a manual microplate (96 microwell plates, Nunc, Roskilde, Denmark) method. Serum was incubated with haemoglobin (Hb) (0.12 mg/mL bovine haemoglobin (Sigma Aldrich, St Louis, USA) in 0.15 M NaCl (Merck Schuchardt, Hohenbrunn, Germany)) leading to preserved peroxidase activity of the complex. By addition of a peroxidase substrate (chromogenic solution; 0.5 M citrate buffer pH 3.8 (0.5 M sodium citrate dihydrate (J.T Baker B.V., Deventer, Holland), 0.5 M citric acid-1-hydrate (Merck)), 1% Tween 20 (Merck), 20 mM phenol (International Biotechnologies Inc., Eastman Kodak Co. Rochester, NY), 0.39 mM dithioerythritol (Sigma), 1.6 mM 4-aminoantipyrine (Sigma), 1 mM 8-anilino-1-naphthalene sulphonic acid (Sigma), and 1 *μ*L 30% H_2_O_2_/0.7 mL solution (Merck)), the activity, which is directly proportional to the amount of haptoglobin in the samples, was measured at 600 nm (SpectraMax M2 Multi-detection Microplate Reader, Molecular Devices, Sunnyvale, Calif, USA) and compared with a haptoglobin standard (2 mg/mL) (Tridelta Development Ltd., Maynooth County Kildare, Ireland).

### 2.14. Multiple Cytokine Assays

For quantitative analysis of cytokines (interleukin-(IL-) 1*β*, IL-4, IL-6, IL-10, IL-12, IL-17, IL-18, TNF-*α*, IFN-*γ*) and leptin, Milliplex microbeads array system was used following the manufacturer's recommended protocols. All samples were run in duplicates, and the results were evaluated by use of MilliplexTM Analyst v. 3.4 (Millipore). Values just below the standard curves were set at the value of detection limit.

### 2.15. Statistics

**Figure 1:**
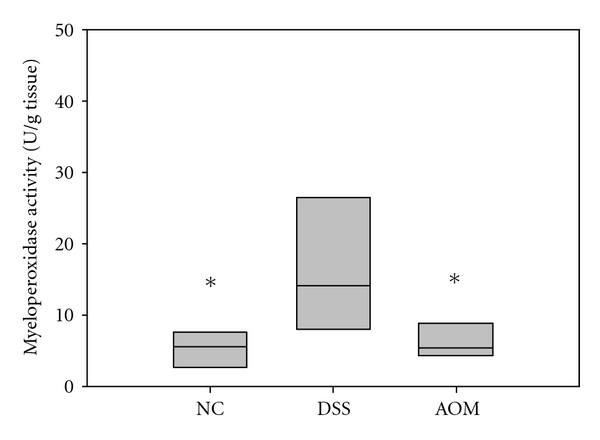
Myeloperoxidase activities (U/g tissue) in colonic tissue; *denotes *P* < 0.05 compared to the DSS group.

Body weight change, DAI scores, MPO activity, number of dysplastic lesions, number of ulcers, scoring used for histopathologic evaluation of colonic and liver samples, lactobacilli and *Enterobacteriaceae* counts, haptoglobin, cytokines, and leptin (Figures 1, 9, and 10 and Tables 2 and 3) were presented as medians with 25 and 75 percentiles. The statistics were conducted in SigmaStat version 3.0 (SPSS Inc., Chicago, Ill, USA). Differences between all groups were evaluated by Kruskal-Wallis test one-way ANOVA on ranks followed by all pairwise multiple comparison procedures (Student-Newman-Keuls method) if appropriate. The differences between treatment groups were assessed by a Mann-Whitney rank sum test. The correlation between expectations of benefit was ascertained using Pearson's correlation coefficient. Calculation of the incidence of steatosis, inflammatory cell infiltration, stasis, loss of parenchyma, and translocation (Table 3) was conducted in QuickStat version 2.6 and was evaluated by the Fisher exact test.

SCFAs were calculated as the concentrations of each acid (*μ*mol/g) multiplied by the caecal amount. The proportion of individual SCFAs was calculated as a percentage of total SCFAs for each rat before statistical evaluation (Table 5). Feed intake, caecal content, caecal tissue weight, caecal pH, and SCFAs were presented as means ± SEM (standard error of the mean). For statistical evaluation of the differences between samples, one-way ANOVA using the general linear model procedure (GLM, ANOVA) was used (Table 5). The Minitab statistical software (Release 14) was used to make these evaluations. Levels of significance were tested at *P* < 0.05 unless stated otherwise.

## 3. Results

### 3.1. Feed Intake and Body Weight Change

At the start of the specialised regimens, there was a body weight difference between the groups, with a slightly higher bodyweight in the AOM group (209 (204.5–210.8) g/animal) compared with the DSS group (189.5 (181.5–191.0) g/animal) and the NC (189 (187.0–192.0) g/animal) group (*P* < 0.001).

During the study, the feed intake in the NC group (20.1 g/(d and rat)) and the DSS group (19.2 g/(d and rat)) was similar, while the intake in the AOM group was somewhat lower (17.1 g/(d and rat)). All animals gained weight during the study, and, at the end, the body weight change in the NC group (240.5 (218.5–265.5) g/animal) was higher than in DSS (173.5 (167.0–217.5) g/animal, *P* = 0.015) or AOM (159.0 (152.3–178.0) g/animal), *P* < 0.001) groups. When feed consumption was taken into account, a significant body weight change (g/kg feed/animal) was found between the NC (72.9 (66.2–80.5) and the AOM group (56.8 (54.4–63.6) (*P* = 0.014)) but not compared to the DSS group (56.0 (53.9–70.2)). No differences in caecal content or caecal tissue weight were found between the groups (data not shown).

### 3.2. Disease Activity Index

The disease activity index (DAI) after the first DSS cycle of the DSS group (4% DSS; 0.33 (0.33–0.5)) did not significantly differ from the DAI of the AOM group after the singular treatment with 5% DSS (0.67 (0.33–0.67)). Many animals had soft stools and were haemoccult positive, but their body weight gains were not decreased during this period of treatment. Thereafter, only mild clinical symptoms (slight rectal bleeding and soft stool) were occasionally noted in the AOM group. In the DSS group, the signs of colitis gradually disappeared during the pure water period after the 1st cycle. During the second cycle, the DAI score reached significant difference (1.33 (0.67–1.67) *P* = 0.01) and over time the score gradually increased and did not revert between the cycles of DSS administration. At the end of the experimental period, all animals in the DSS group exhibited rectal bleeding, loose stool, and body weight loss at the end of DSS cycles and a significant higher DAI score was observed between the first and eleventh cycle of DSS administration (2.0 (1.5–2.3) *P* < 0.001). The mortality rate was 0%.

### 3.3. Myeloperoxidase Activity

Myeloperoxidase activity which was used to quantify neutrophil accumulation in colonic tissues was significantly (*P* < 0.05) higher in the DSS group (14.1 U/g (8.8–20.9)) than in the NC group (5.6 U/g (3.0–5.8)) and in the AOM group (5.5 U/g (4.9–8.7)) (Figure 1).

### 3.4. Histological and Macroscopic Alterations of Colon

**Figure 2:**
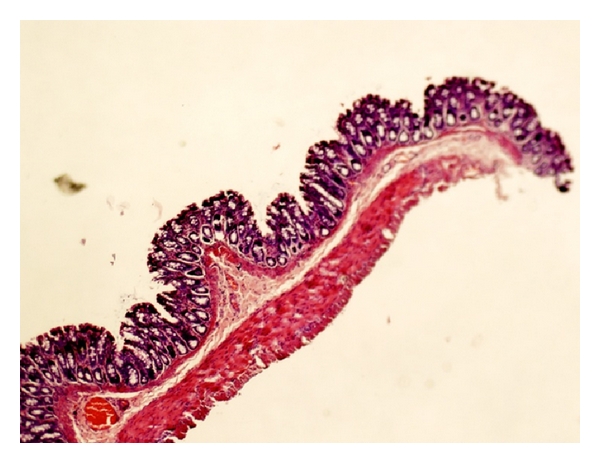
Normal crypt architecture and absence of inflammation seen in colonic mucosa from a rat in the NC group.

The mucosal architecture of the NC group was assessed to be normal. There was no ulceration of the epithelial lining, and the crypts and the lamina propria inflammatory infiltrate were normal (Figure 2). 

**Figure 3:**
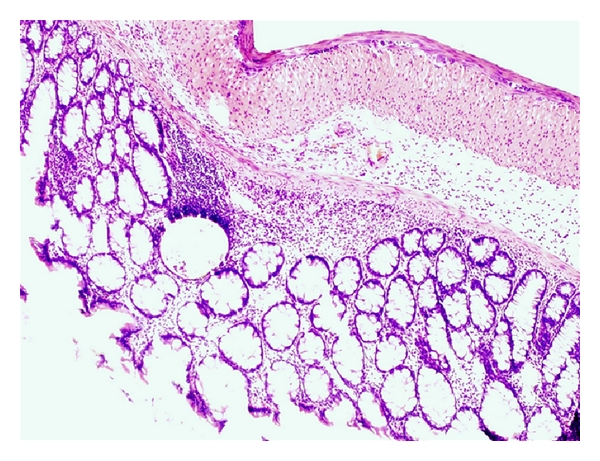
Flat dysplasia seen in colonic mucosa from a rat in the DSS group.

**Figure 4:**
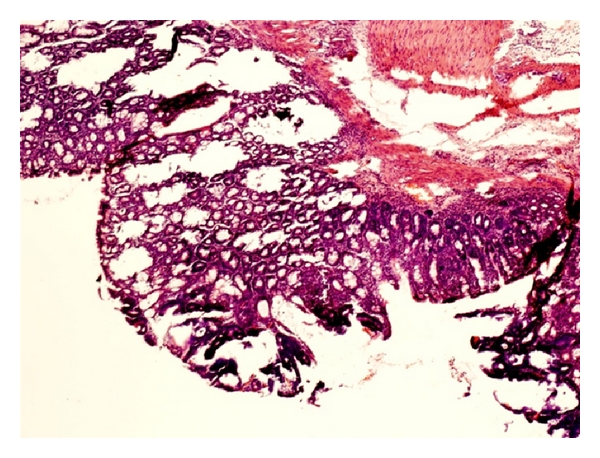
Dysplastic lesion in chronic inflamed colonic mucosa from a rat in the DSS group.

**Figure 5:**
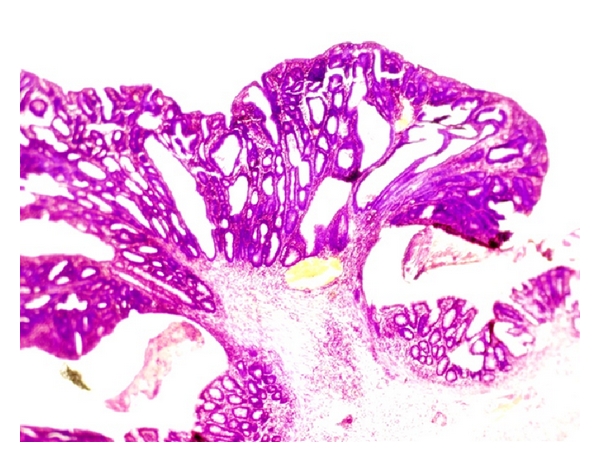
Pedunculated adenocarcinoma with fibrovascular stalk and heads containing dysplastic epithelial glands in colonic mucosa from a rat in the AOM group.

**Figure 6:**
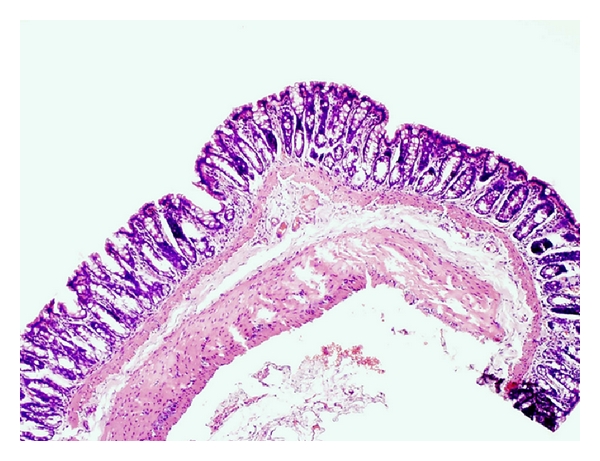
Colonic mucosa free from dysplastic foci in the area between the polyps in the AOM group.

In light microscope, the DSS group showed colonic inflammation mostly confined to the mucosa and submucosa, with loss of surface epithelium, inflammatory cell infiltrations, loss of goblet cells, crypt distortion and abscesses, mucosal ulceration and erosion, and accompanying submucosal edema. The diseased condition seemed to be distributed throughout the colon but was particularly prominent on the left side of the large intestine and transverse colon. Regenerative and hyperplastic epithelium, which morphologically mostly resembled low-grade dysplasia with some sections of high-grade dysplasia and polyps diagnosed as adenocarcinomas, was observed (Figures 3 and 4). The incidence of low-grade dysplasia was significantly higher in the DSS group than in the NC group and the AOM group (Table 2, *P* < 0.05). In the AOM group, several features of high-grade dysplasia was found with occasional pedunculated adenocarcinomas showing fibrovascular stalks and heads containing abundant dysplastic epithelial glands (Figure 5), but compared to the other groups, the difference was not significant. In contrast to the DSS group, the polyps in the AOM group were segregated by minimal inflammation, erosion, or hyperplastic epithelium, indicating that the disease process was not continuous (Figure 6). Examination of specific histologic scores showed no significant difference (Table 2).

**Figure 7:**
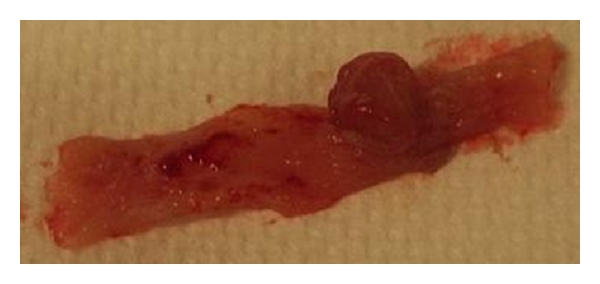
Ulceration, thickening of the mucosal wall, and a polyp in distal colon from a rat in the DSS group.

**Figure 8:**
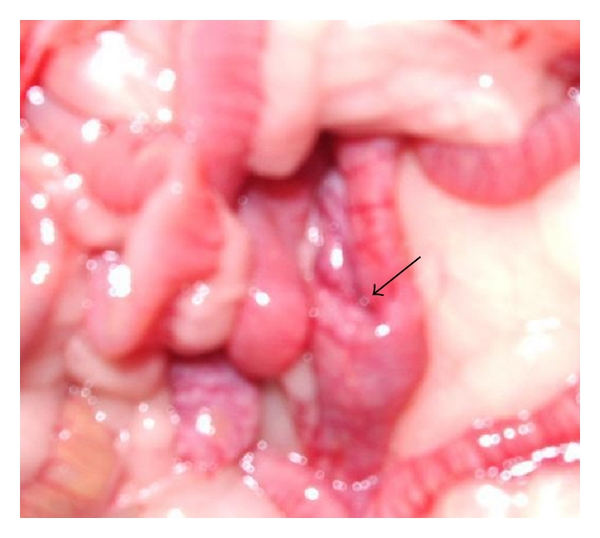
Invagination as a cause of a polyp in colon of a rat in the DSS group.

**Figure 9:**
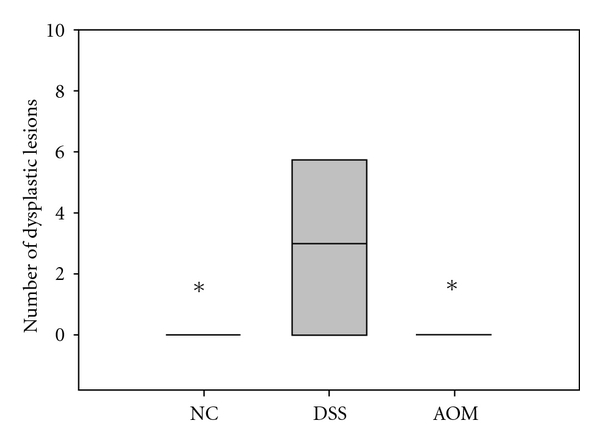
Number of dysplastic lesions in colon classified as low-grade dysplasia in different treatment groups. DSS 3.0 (0.0–5.5); AOM 0.0; NC 0.0. *Denotes *P* < 0.05 compared to the DSS group.

**Figure 10:**
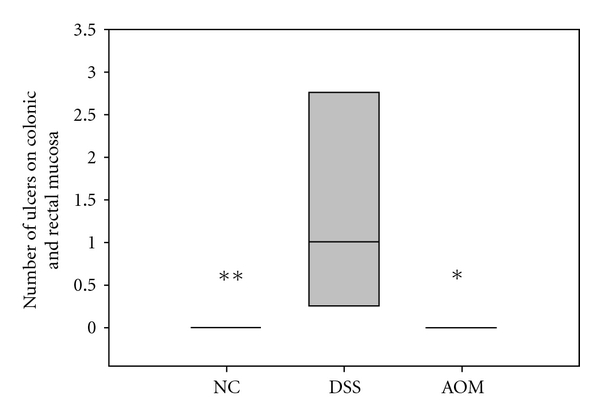
Number of ulcers of colonic and rectal mucosa in different treatment groups. DSS 1.0 (0.5–2.5); AOM 0.0; NC 0.0. **Denotes *P* ≤ 0.01 and *denotes *P* < 0.05 compared to the DSS group.

Morphological examination of colon from each animal revealed visible thickening of the colon wall in the DSS group (Figure 7). Invaginations as a cause of polyps (Figure 8) and dilated descending colon were occasionally seen in both the DSS group and the AOM group. There was no sign of gross mucosal ulceration or thickening of the colon wall in the AOM group. Quantitatively, the number of lesions classified as low-grade dysplasia (3.0 (0.0–5.5)) was significantly higher in the DSS group compared to the NC group (0.0 (0.0-0.0); *P* = 0.038) and AOM group (0.0 (0.0-0.0); *P* = 0.04) (Figure 9). A total of 26 dysplastic lesions distributed over 5 animals were found in the DSS group compared to none in the other groups. The same pattern was achieved for colonic ulcers, that is, 11 ulcers were found in the DSS group (1.0 (0.5–2.5), while none were found in the other two groups (*P* = 0.01 versus NC group; *P* = 0.014 versus AOM group) (Figure 10). The number of polyps in the AOM group was in total 30, distributed over 3 animals. In the DSS group, 3 polyps were found, distributed over 2 animals. No polyps were found in the NC group.

### 3.5. Translocation and Histopathological Evaluation of the Liver

No significant difference in the incidence of translocation of live bacteria to the liver or to the mesenteric lymph nodes was seen between groups (Table 3).

**Figure 11:**
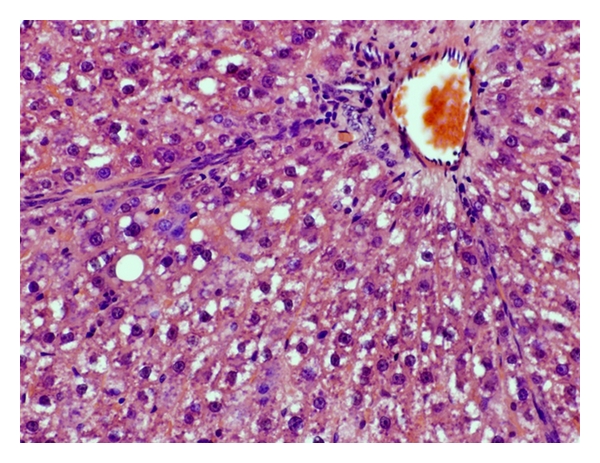
Macrovesicular steatosis, involving most regions of the hepatic lobule. The intracytoplasmic fat is seen as white vacuoles in a rat of the NC group.

**Figure 12:**
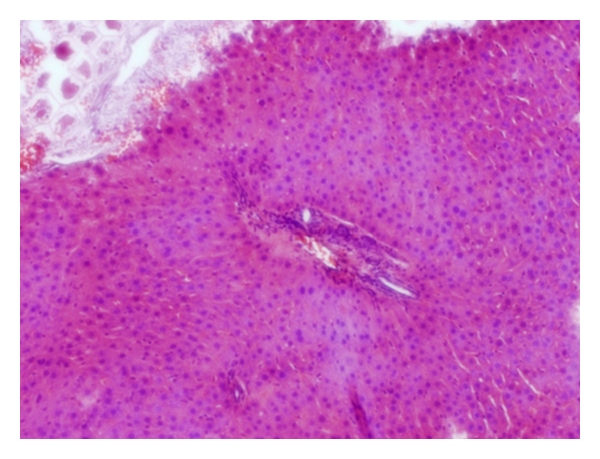
Focal areas with parenchymal loss and haemorrhage in the liver from a rat of the DSS group.

**Figure 13:**
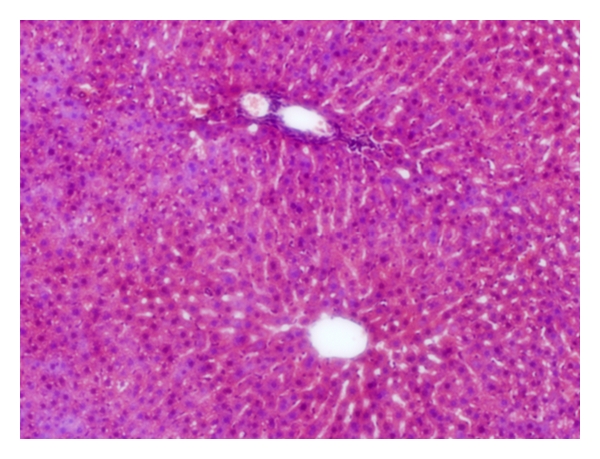
Slightly hepatic steatosis in the liver of a rat in the AOM group.

Livers of the NC group showed varying degree of macrovesicular steatosis (Figure 11). Mild steatohepatitis was observed in one case within the steatotic areas. In the DSS group, a mild-to-moderate degree of steatosis was found. Liver lobules had occasional focal areas with parenchymal loss, haemorrhage, and small inflammatory infiltrations in nonsteatotic area (Figure 12). Only slight hepatic steatosis could be seen in specimens from the AOM group. In neither case, parenchymal inflammatory infiltration, parenchymal loss, or haemorrhage could be found (Figure 13). 

By use of a numerical scoring system, the degree of parenchymal inflammatory infiltration in nonsteatotic area was shown to be significantly higher in the DSS group (2.0 (2.0-2.0)) compared to the NC group (0.0 (0.0-0.0) *P* < 0.001) and AOM group (0.0 (0.0-0.0) *P* < 0.001) (Table 3). The incidence of parenchymal inflammatory infiltration was also found to be significantly higher in the DSS group (*P* < 0.001) as well as stasis (*P* < 0.05) and a significantly lower incidence of steatosis in the AOM group compared to the other groups (*P* < 0.05) (Table 3).

### 3.6. Feacal Bacterial Counts

At the point in time corresponding to the 7th day of DSS administration (first DSS cycle), the faecal count of *Enterobacteriaceae *increased, reaching levels of log 8.6 (8.4–8.9) CFU/g (AOM) (*P* < 0.001) and 7.4 (7.1–7.7) CFU/g (DSS) (*P* = 0.001), compared to the base line. The difference of the *Enterobacteriaceae* count between the AOM group and the DSS group was significant (*P* < 0.001). At the last day of the study, the *Enterobacteriaceae* count was still higher in the DSS group compared to base line (7.6 (7.5–7.8)) CFU/g (*P* < 0.001), but not in the AOM group (6.5 (6.1–6.8) CFU/g. The load of *Enterobacteriaceae* in the DSS group was significantly higher than both the NC group (*P* = 0.001) and the AOM group (*P* < 0.001).

After 7 d of DSS treatment at the first cycle, faecal viable count of lactobacilli was higher in the AOM group (11.2 (11.1–11.5)) CFU/g than in the DSS group (10.0 (9.1–10.3)) CFU/g (*P* < 0.001). The AOM group also showed an increase compared to the start of the study (9.5 (9.1–9.8)) CFU/g (*P* < 0.001). From the start to the end of the study, only the NC group exhibited an increase in lactobacilli count (start 10.0 (9.2–10.1) CFU/g; end 10.4 (10.2–10.8)) CFU/g (*P* = 0.006), and, from faecal samples collected at the last day of the study, the concentration was higher in the NC group than in the other two groups (DSS, 9.1 (9.0–9.2) CFU/g; AOM, 9.2 (9.1–9.3) CFU/g, *P* < 0.001).

### 3.7. Identification of Faecal Lactobacilli and Translocating Bacteria in the Liver

#### 3.7.1. Faeces

Not all of the picked isolates from the Rogosa plates could be identified. A total of 30 out of 44 picked colonies were identified through RAPD band pattern comparison and 16S rDNA sequencing. Obtained sequences were at least 700 base-pair long, and the results showed no less than 99% sequence similarity to their nearest database entries. The majority of the recovered sequences were identified to different *Lactobacillus* spp., only a single sequence was assigned to *Bifidobacterium *(Table 4). 

In the NC group, *B. animalis, L. reuteri, and L. murinus* were identified during the baseline period. At the point in time corresponding to the 10th DSS cycle, *L. murinus* was dominant. In the DSS group *L. murinus* dominated through the whole study period. In the AOM group, *L. reuteri* and *L. intestinalis *were found in the baseline samples. After the first and only DSS administration to the AOM group, *L. reuteri* and *L. gasseri* were isolated, and, at termination, *L. reuteri* and *L. vaginalis* were identified (Table 4). 

#### 3.7.2. Liver

Fifteen bacterial isolates from the liver were subjected to 16S rDNA sequencing. Only, *Lactobacillus animalis* was identified from livers in the NC group (Table 3). In the DSS group, *Kocuria rhizophila*, *Micrococcus luteus*, *Clostridium ramosum*, and *Staphylococcus warneri* were found besides different *Lactobacillus* spp., and, in livers from the AOM group, only *Clostridium perfringens* and *K. rhizophila* could be identified (Table 3).

### 3.8. SCFAs in the Hindgut

No differences in caecal pH were found between the groups. The NC group had a higher caecal level of butyric acid (26.7 *μ*mol/g) than the DSS group (12.1 *μ*mol/g, *P* = 0.001) or AOM group (16.7 *μ*mol/g, *P* = 0.048). In the distal part of colon, the levels of acetic and propionic acid in the DSS group (47.2 *μ*mol/g and 13.1 *μ*mol/g) were higher compared with the NC group (31.7 *μ*mol/g and 8.9 *μ*mol/g) (*P* = 0.002 and *P* = 0.018, resp.) (data not shown).

The proportion of acetic acid was higher, and that of butyric acid was lower in the DSS group than in the NC group (*P* = 0.007 and *P* < 0.001, resp.) (Table 5). Similar differences could also be found in the proximal (*P* = 0.007 and *P* = 0.018, resp.) and distal part of colon (*P* < 0.001 and *P* = 0.002, resp.). Furthermore, the proportion of butyric acid in the proximal colon in the DSS group was lower than that in the AOM group (*P* = 0.047). In the distal part of colon in the AOM group, the acetic acid proportion was higher than in the NC (*P* = 0.035) (Table 5).

### 3.9. SCFAs in Aortic Blood

Acetic acid was the main acid in the aortic blood in all rats (94.3%), followed by butyric acid (2.0%), i-valeric acid (1.5%), i-butyric acid (1.1%), and propionic acid (1.0%) (data not shown).

The level of propionic acid was higher in the DSS group (11.2 *μ*mol/L, *P* < 0.001) than in the AOM group (7.9 *μ*mol/L, *P* = 0.002), but both were lower than the level in the NC group (15.4 *μ*mol/L, *P* = 0.039). The same relationships were found when evaluating the proportions. The AOM group had a lower proportion of propionic acid (0.7%) compared with the NC group (1.3%, *P* = 0.022) or DSS group (1.1%, *P* = 0.039).

### 3.10. Body Temperature

The baseline temperature of the rats was 37.4°C (37.0–37.5) in the NC group, 36.8°C (36.5–37.0) in the DSS group, and 37.2°C (36.6–37.3) in the AOM group, with a significant difference found between the NC group and the DSS group (*P* = 0.014). The temperature varied over time, and, during the corresponding point in time of the fifth DSS cycle, the temperature was again lower in the DSS group (36.8°C (36.2–37.3)) than in the AOM group (37.6°C (37.3–38.3)) (*P* = 0.014). At the end of the study (corresponding to the tenth DSS cycle), the temperature reached similar values in all three groups (NC, 37.6°C (36.9–37.7)); DSS, 37.5°C (36.6–37.7); AOM, 37.5°C (37.1–37.8).

### 3.11. Haptoglobin

The haptoglobin baseline value of the DSS group (0.38 mg/mL (0.26–0.55) was higher than for the other groups (NC group, 0.17 mg/mL (0.14–0.35); *P* = 0.043 compared with the DSS group, and AOM group, 0.17 mg/mL (0.06–0.23); *P* = 0.006 compared with the DSS group). On the seventh day of DSS administration (1st cycle), the haptoglobin level of the DSS group was significantly lower (*P* = 0.041) than for the AOM group (DSS group, 0.26 mg/mL (0.23–0.38); AOM group, 0.49 mg/mL (0.45–0.49)). At the points in time corresponding to the 5th through 10th DSS cycles, no differences were seen between the groups. Over time (from start to the 10th DSS cycle), the haptoglobin levels increased in all three groups, NC 0.73 mg/mL (0.47–0.90) (*P* < 0.001), DSS 0.75 mg/mL (0.51–0.98) (*P* = 0.029), and AOM 0.62 mg/mL (0.57–0.65) (*P* < 0.001). At the point in time corresponding to the tenth DSS cycle, a negative correlation was found between body temperature and haptoglobin levels, where the temperature tended to decrease while the values of haptoglobin increased (*r* = −0.50, *P* = 0.02).

### 3.12. Multiple Cytokine Assays

No differences between the groups were found in terms of the cytokine profile (data not shown). In the end of the study, the level of leptin in aortic blood was lower in the DSS group (3463.35 (2755.60–4678.69) pg/mL) than in the NC group (7012.10 (4440.81–8519.44 pg/mL) (*P* = 0.04)) and the AOM group (5638.15 (4806.53–6588.72) (*P* = 0.038)).

## 4. Discussion

A procedure of repeated cyclic administration of 4% DSS in the drinking water followed by periods without DSS was used for inducing chronic inflammation, dysplasia and cancer and was compared to a model using AOM-induced colonic carcinogenesis. DSS is not considered to be mutagenic and responds negatively in the Ames test for mutagens [29]. Consequently, the changes occurring in the DSS group can presumably be attributed to the inflammation and regeneration of the colonic mucosa, recurrence-remission cycles typical of clinical ulcerative colitis cases [30]. In contrast, AOM is a genotoxic colon carcinogen, in this case given once followed by a short exposure of DSS for promotion of colitis-associated colon cancer. This AOM model has been implicated to generate large bowel adenocarcinomas in the short term, and their histology and biological alteration resemble those found in humans [31]. However, usage of AOM alone induces polypoid tumours with clinical, histological, and molecular features that mimic human sporadic colon cancer [32].

DSS feeding for seven days resulted in a mild colitis in both treatment groups, and this status continued during the experimental period for some of the animals in the AOM group. A gradually increased inflammatory activity was recorded in the DSS group, indicated by a significant increase in the disease activity index. The impaired recovery between exposures to DSS may suggest a chronologic sequence, where repeated uninhibited acute inflammatory responses develop chronicity. The analysis of body weight gain across the experimental period revealed a lower body weight gain for both the DSS and AOM group.

MPO content is a marker of neutrophil infiltration at the site of mucosal injury, and assessment of MPO activity is considered a reproducible and qualitative estimate of mucosal inflammation [33], so it may serve as a quantitative index of disease severity. Analysis of colonic MPO activity showed an elevation in the DSS group (Figure 1), indicative of severe mucosal inflammation of descending colon. MPO levels have also been found to be increased in colorectal mucosa of patients with adenoma or carcinoma [34]. 

The pathogenesis of colorectal carcinogenesis associated with colonic inflammation is believed to involve progression from inflamed and hyperplastic cryptal cells, through dysplasia, to adenoma and carcinoma [35]. Microscopic findings of descending colon demonstrated histological abnormalities with dysplasia and adenocarcinomas in both the DSS group and the AOM group (Figures 3, 4, and 5). However, the incidence of low-grade dysplasia was significantly elevated by repeated administration of DSS (Table 2). This finding corresponds with the frequency of appearance of the number of low-grade dysplasia (Figure 9) and mucosal ulcers (Figure 10) found through macroscopic observation. By using DSS and different DAI scores, Cooper et al. [14] were able to demonstrate that animals with dysplasia and/or cancer have higher inflammatory scores. As precursors of adenomas, dysplasia has been identified as a hallmark of malignant potential [36]. Although not significantly different, a high number of adenocarninomatous polyps with severe dysplasia were found in the AOM group, distributed in few animals. Obviously, animals from the same group at the same time points may or may not develop carcinogenesis, despite equivalent treatment. This diversity of diagnosis could be explained by differences in host immune system and individual genotype [37]. Strain differences in the susceptibility to both AOM and DSS have been found in mice [38], and it seems that Sprague-Dawley rats in their response to AOM are more prone to individual animal variations than during cyclic DSS treatment. Macroscopically, no distinct signs of mucosal inflammatory reactions such as thickening of the mucosal wall or gross mucosal ulceration were observed in the AOM group. Mild mucosal redness or minor ulcers might cause the slight rectal bleeding, found in a few animals. Aside from the polyps, there were no dysplastic foci in the mucosa in this group (Figure 6), probably because inflammation was less intense or resolved more rapidly after one cycle of DSS exposure. 

There is a close relationship between UC and various hepatobiliary disorders [10], and, during colonic cancer, malignant tissue may disrupt the bowel architecture and increase gut permeability [6]. Through the portal vein, gut-derived components are easily accessible to the liver. Increased translocation through the intestinal epithelium during UC may allow bacterial antigens and toxins to cause inflammatory reactions when reaching the liver [39]. In the present study, inflammatory infiltrations in the parenchyma were neither seen in the NC nor the AOM group but were prominent in the DSS group (Table 3, Figures 11, 12, and 13). Moreover, the incidence of stasis was also elevated in the DSS group (Table 3). The reason for the stasis seen in liver specimens is unclear, but theoretically it could be due to circulatory failure. We do not know if the DSS treatment causes other damages, for instance to the heart or the kidneys. The occurrences of steatotic areas were less frequently found in the AOM than in the NC or DSS group (Figures 11, 12, and 13, Table 3). Fatty infiltration of hepatocytes has been reported during intestinal inflammation [40]; however, the colonic mucosa was not inflamed in the NC group but was in the DSS group. It seems as the inflamed condition in the livers of the DSS group has proceeded past the stage of steatosis. 

Although no significant difference in the incidence of translocation was found between groups in the present study, no potential pathogenic bacteria were found in the livers of the NC group (Table 3). Of the lactobacilli found, *L. animalis* (phylogenetically related to *L. murinus*), *L. frumenti* and *L. antri *(members of the *L. reuteri* subgroup) [41], and *L. gasseri* are all normally found in rodents [42, 43]. Neither *S. warneri* nor *M. luteus* is regarded as a dominating part of the resident bacterial flora of the gut, but *S. warneri* has been isolated from the skin of laboratory mice [44], and *M. luteus* has been shown to colonise the gastrointestinal tract of rats after inoculation [45]. Both *M. luteus* and *K. rhizophila* are able to cause infections in humans [46, 47]. In this study, the chronicity of the debilitating disease and the accompanying inflammation may put the animals at increased risk for opportunistic infections. *C. ramosum* and *C. perfringens* were isolated from the DSS group and the AOM group, respectively (Table 3). Different species of *Clostridium* have been implicated in the induction of intestinal inflammation and may be detrimental when a dysfunction of the colonic mucosal barrier is present [48]. Okayasu et al. [30] showed increased number of different members of the families *Enterobacteriaceae*, *Bacteroidaceae,* and *Clostridiaceae* during DSS-induced colitis, and increasing numbers of *B. distasonis* and *C. ramosum* were particularly significant after repeated administration. Enhanced antibody response against *C. ramosum*, which was one of the most frequently isolated anaerobes from the inflamed mucosa of UC patients, has been demonstrated [49]. No members of the family *Bacteroidaceae* were found in the livers in the present study. Viable count of faeces revealed an increase in *Enterobacteriaceae* after 11 administration cycles of the alternating DSS-tap water regime when compared with preadministration values for the DSS group only. This value was also significantly higher compared to the other two groups. Faecal microbial communities in patients with UC differ from those in healthy individuals [7]. An overrepresentation of *Escherichia coli* (belonging to the family *Enterobacteriaceae*) with high metabolic activity, along with lower numbers of lactobacilli has been seen [50, 51]. This is comparable to our results, which also showed a decrease of lactobacilli in both the DSS and the AOM group compared to the NC group. 

Colonic microbial communities and SCFA fermentation in patients with colonic disease might differ from the ones considered as normal. Significantly higher proportions of acetate and lower proportions of butyrate in enema samples from patients with adenomatous polyps have been shown [52]. These results suggest an increase in the floral capacity to produce acetate and a decrease in the capacity to form butyrate during colon cancer [52]. Furthermore, it has been reported that the capacity of the faecal microbiota from patients with colonic adenomas and colon cancer to produce butyrate was significantly reduced [53]. Another hypothesis could invoke differences in the metabolism of SCFAs by the colonocytes [52]. In the present study, significantly higher proportions of acetate and lower proportions of butyrate in the content of caecum, proximal, and distal colon were found in the DSS group compared to the NC group (Table 5). Furthermore, as in the study of Weaver et al. [52], the concentration of acetate and propionate were significantly higher in distal colon of the DSS group; thus, the results from the analysis of luminal SCFAs indicate similarities between the model of DSS-induced tumour development and the clinical situation.

In aortic blood, the concentration of propionic acid was higher in the DSS group compared to the AOM group, but both were lower than the NC group. It has been speculated whether a cirrhotic, dysfunctional liver is able to metabolise SCFAs, leading to a rise in systemic concentrations [54]. Propionate serves as a substrate for gluconeogenesis [54], and since the livers in the AOM group were the less affected and because this group also stated the lowest feed intake and body weight gain, it may indicate a higher gluconeogenic rate due to starvation caused by occlusive colon cancer. 

Haptoglobin, an acute phase reactant, has been implicated as a useful marker of inflammation in rats [55] and is elevated during DSS administration [56]. After seven days of DSS exposure in the first and only DSS cycle of the AOM group, a higher haptoglobin level was found in this group than in the DSS group, probably as a result of the higher concentration of DSS. At the end of the experimental period, no difference was found between the three groups. It might have been expected that the haptoglobin level in the heavily inflamed DSS group would have been higher than that of the NC group. On the other hand, the NC group had fattening tendencies which can explain a somewhat increased haptoglobin level, while it is well known that liver injury results in decreased haptoglobin concentrations [57, 58], that is, both inflammation due to colitis and due to fattening tended to increase the haptoglobin level, and presumably to the same degree in the present case.

In the serum, only very low levels of cytokines were recorded (data not shown). The cytokine production profile for this model in rats has not been established, but chronic colitis induced by DSS in mice displayed an enhanced prohumoral cytokine bias [59], and so these results are unexpected and have to be further elucidated.

Serum leptin is directly correlated with the body fat stores, increasing with fat accumulation [60], and the highest level was found in the NC group. Evidence has demonstrated that the level of haptoglobin was increased in obese mice [56], and since both leptin and haptoglobin were increased in the NC, this might presumably indicate overweight animals in this group, which also corresponds to the liver steatosis. Most likely, there is a match between age, low physical activity, and increased body weight. The lowest level was found in the DSS group, which coincides with findings from UC patients, suggesting that chronic intestinal inflammation may decrease circulation leptin values [61].

## 5. Conclusions

Chronic colitis associated with dysplasia has been found in rats after DSS exposure [62]. In the present study, we were able to develop low-grade as well as high-grade dysplasia and colitis-associated cancer. As seen in UC patients [2], development of dysplastic lesions seems to be related to the duration and severity of the colonic inflammatory processes. Our colitis model using repeated exposure to DSS needs a longer treatment period to induce carcinogenesis, but the AOM model appears to show dissociation between the severity of intestinal inflammation and development of cancer. In this DSS model, we were also able to document altered liver function by endogenous inflammatory mediators, as well as fermentation patterns that closely mimic the clinical situation, and so the model may be a valuable tool for investigation of UC-related dysplasia and colon cancer.

## Figures and Tables

**Table 1:** Diet composition. Composition of test diets (g/kg dwb) given to rats in all groups (NC, DSS, and AOM).

Component	
Oat bran	291^1^
Casein	160
DL-methionine	1.2
Maize oil	50
Mineral mixture^2^	48
Vitamin mixture^3^	8
Choline chloride	2
Sucrose	100
Wheat starch^4^	380

^1^Corresponding to 50 g dietary fibre/kg diet (dwb).

^2^Containing (g kg^−1^) 0.55 CuSO_4_·H_2_O, 2.0 ZnSO_4_·7H_2_O, 498 KH_2_PO_4_, 258 NaH_2_PO_4_·2H_2_O, 487 CaCO_3_, 0.1 KI, 86 MgSO_4_, 12 FeSO_4_·7H_2_O, 5 MnSO_4_·H_2_O, 0.03 CoCl·6H_2_O, 153 NaCl, 0.02 CrCl_3_·6H_2_O, 0.02 Na_2_Se.

^3^Containing (g kg^−1^) 0.62 menadion, 2.5 thiamin hydrochloride, 2.5 riboflavin, 1.25 pyridoxin hydrochloride, 6.25 calcium pantothenate, 6.25 nicotinic acid, 0.25 folic acid, 12.5 inositol, 1.25 p-aminobenzoic acid, 0.05 biotin, 0.00375 cyanocobalamin, 0.187 retinol palmitate, 0.00613 calciferol, 25 d-*α*- tocopheryl acetate, and 941.25 maize starch.

^4^Wheat starch (Cerestar, Krefeld, Germany).

**Table 2:** Histological evaluation. Histological evaluations of colonic samples. Number of animals showing the histopathologic lesion per total number analysed samples in the group and an index calculated on the basis of the scoring system.

	Low-grade dysplasia	High-grade dysplasia	Index
NC	0/8	0/8	1.0
DSS	4/8*	1/8	1,75
AOM	0/7	3/7	1.86

*Denotes *P* < 0.05 compared to the NC and AOM group.

**Table 3:** Liver injury. Liver status of different treatment groups of rats expressed as scoring values (degree of scoring: received value/maximum value) and incidence of phenomena/total number of animals. Percentages of the values are shown in parentheses. Incidence of translocation is mentioned in the following order: growth on Rogosa agar; Brain Heart Infusion agar (incubated anaerobically); Brain Heart Infusion agar (incubated aerobically). Fifteen isolates from the liver were subjected to 16S rDNA sequencing, and the similarity level was ≥99% for twelve of the samples, and 98.6% for one of the *L. animalis* (DSS group), 95.6% *L. frumenti *(*antri*) (DSS group), and 93.1% for one of the *L. antri *(DSS group). If the species is identified for more than one sample, the number is shown in parentheses.

Scoring values							Incidence						
Groups	Degree of steatosis	Cell infiltration around CV within steatotic areas	Cellinfiltration elsewhere in the parenchyma	Stasis	Loss of parenchyma		Steatosis	Cell infiltration around CV within steatotic areas	Cell infiltration elsewhere in the parenchyma	Stasis	Loss of parenchyma	Translocation	Species designation of translocated bacteria

NC	10/21 (47.6%)	2/21 (9.5%)	0/21***	0/21	0/21		7/7	1/7	0/7***	0/7*	0/7	0/8; 1/8; 1/8	*L. animalis*
DSS	10/24 (41.7%)	2/24 (8.3%)	16/24 (66.7%)	8/21 (38.1%)	6/18 (33.3%)		8/8	1/8	8/8	4/7	3/6	3/8; 2/8; 4/8	* L. animalis *(2), * L. apodemi, * *K. rhizophila, * * L. frumenti * * (L. antri*), *L. antri *(2), * L. gasseri, * * M. luteus*, * C. ramosum, * * S. warneri *(2)
AOM	4/18 (22.2%)	0/18	0/18***	0/18	0/18		2/6^#^	0/6	0/6***	0/6*	0/6	0/7; 1/7; 2/7	* K. rhizophila* * C. perfringens *

Scoring values: ***Denotes *P* < 0.001 compared to the DSS group.

Incidence: ***Denotes *P* < 0.001 and *P < 0.05 compared to the DSS group, ^#^ *P* < 0.05 compared to the NC and DSS group *Lactobacillus animalis* (*L. animalis*), *Lactobacillus apodemi* (*L. apodemi*), *Kocuria rhizophila* (*K. rhizophila*), *Lactobacillus frumenti* (*L. frumenti*), *Lactobacillus antri* (*L. antri*), *Lactobacillus gasseri* (*L. gasseri*), *Micrococcus luteus* (*M. luteus*), and *Clostridium ramosum* (*C. ramosum*), *Staphylococcus warneri* (*S. warneri*), *Clostridium perfringens *(*C. perfringens*). Species shown in parentheses have the same sequence similarity as the previous species.

**Table 4:** Faecal flora. Identification of isolates from plate count of faeces from different rat groups (*L.* = *Lactobacillus* and *B*. = *Bifidobacterium*).

Group	Base line	Time point after DSS cycle 1	Time point after DSS Cycle 10	Termination
NC	*B. animalis * *L. reuteri * * L. murinus*		*L. murinus * *L. murinus * *L. murinus*	
DSS	*L. murinus * *L. murinus * *L. murinus * *L. murinus*	*L. murinus * *L. murinus * *L. murinus*	*L. murinus * *L. murinus * *L. murinus * *L. murinus*	*L. murinus * *L. murinus * *L. murinus*
AOM	*L. reuteri * *L. intestinalis * *L. intestinalis*	*L. gasseri * *L. reuteri * *L. gasseri*		*L. vaginalis* * L. vaginalis * *L. vaginalis* * L. reuteri*

**Table 5:** Short-chain fatty acids. Distribution between acetic, propionic, and butyric acid (%) in the gut content of rats in different treatment groups^1,2^.

	Normal		DSS		AOM	
	Mean	SEM	Mean	SEM	Mean	SEM
Caecum^3^						
Acetic	55.1^a^	2.6	66.0^b^	1.4	62.1^a,b^	2.8
Propionic	14.4	0.6	16.9	1.1	14.7	0.8
Butyric	30.5^a^	2.4	17.1^b^	1.1	23.2^a,b^	2.7
Proximal^3^						
Acetic	62.2^a^	1.8	69.5^b^	1.2	65.2^a,b^	1.5
Propionic	17.8	0.9	16.1	1.0	15.5	0.8
Butyric	20.0^a^	1.1	14.4^b^	1.4	19.4^a^	1.6
Distal^4^						
Acetic	57.6^a^	1.5	66.4^b^	1.5	63.7^b^	1.4
Propionic	17.2	1.7	18.4	1.5	17.2	1.3
Butyric	25.2^a^	2.2	15.3^b^	1.6	19.1^a,b^	1.5

^1^Mean values with their standard errors of mean for 8 rats per group.

^2^Mean values with unlike superscript letters in the same line were significantly different (*P* < 0.05).

^3^AOM, *n* = 7.

^4^AOM, *n* = 5.
